# Investigating Serum sHLA-G Cooperation With MRI Activity and Disease-Modifying Treatment Outcome in Relapsing-Remitting Multiple Sclerosis

**DOI:** 10.3389/fneur.2022.872396

**Published:** 2022-05-25

**Authors:** Roberta Amoriello, Roberta Rizzo, Alice Mariottini, Daria Bortolotti, Valentina Gentili, Elena Bonechi, Alessandra Aldinucci, Alberto Carnasciali, Benedetta Peruzzi, Anna Maria Repice, Luca Massacesi, Enrico Fainardi, Clara Ballerini

**Affiliations:** ^1^Department of Clinical and Experimental Medicine (DMSC), University of Florence, Florence, Italy; ^2^Department of Chemical, Pharmaceutical and Agricultural Sciences, University of Ferrara, Ferrara, Italy; ^3^Department of Neurosciences, Drugs and Child Health (NEUROFARBA), University of Florence, Florence, Italy; ^4^Flow Cytometry Diagnostic Center and Immunotherapy (CDCI), Careggi University Hospital, Florence, Italy; ^5^Department Neurology II, Careggi University Hospital, Florence, Italy; ^6^Department of Biomedical, Experimental and Clinical Sciences, University of Florence, Florence, Italy

**Keywords:** multiple sclerosis, natalizumab, serum sHLA-G, cytokines, disease activity

## Abstract

Relapsing-remitting multiple sclerosis (RRMS) is a demyelinating disease in which pathogenesis T cells have a major role. Despite the unknown etiology, several risk factors have been described, including a strong association with human leukocyte antigen (HLA) genes. Recent findings showed that HLA class I-G (HLA-G) may be tolerogenic in MS, but further insights are required. To deepen the HLA-G role in MS inflammation, we measured soluble HLA-G (sHLA-G) and cytokines serum level in 27 patients with RRMS at baseline and after 12 and 24 months of natalizumab (NTZ) treatment. Patients were divided into high (sHLA-G>20 ng/ml), medium (sHLA-G between 10 and 20 ng/ml), and low (sHLA-G <10 ng/ml) producers. Results showed a heterogeneous distribution of genotypes among producers, with no significant differences between groups. A significant decrease of sHLA-G was found after 24 months of NTZ in low producers carrying the +3142 C/G genotype. Finally, 83.3% of high and 100% of medium producers were MRI-activity free after 24 months of treatment, compared to 63.5% of low producers. Of note, we did not find any correlation of sHLA-G with peripheral cell counts or cytokines level. These findings suggest that serum sHLA-G level may partly depend on genotype rather than peripheral inflammation, and that may have impacted on MRI activity of patients over treatment.

## Introduction

Multiple sclerosis (MS) is a heterogeneous, autoimmune, and inflammatory disease of the central nervous system (CNS), characterized by the disruption of myelin. An interplay of immune mediators contributes to MS pathogenesis, with a crucial role of T lymphocytes (1). About 2.8 million people worldwide are affected with MS (2) and 85% of all patients show a relapsing-remitting pattern of MS (RRMS), characterized by relapses interspersed with periods of partial or complete recovery (3). The etiology of MS is currently unknown, but several factors have been attributed to a higher risk to develop the disease. The association with human leukocyte antigens (HLA) genes was widely demonstrated (4, 5), as the haplotype HLA-DRB1^*^1501 in North Europe, the USA, and continental Italy, and DR3 and DR4 in the island of Sardinia (6).

Recently, the non-classical HLA histocompatibility antigen G (HLA-G) has been linked with MS susceptibility, particularly with the RRMS form, in a study performed within the Italian population (7, 8). HLA-G is a non-canonical HLA class I molecule, consisting of a heavy and a light chain, that may exist as membrane-bound or soluble isoforms (9). HLA-G is expressed within a limited variety of tissues, e.g., the thymus and pancreas (10, 11). Furthermore, it was identified on placental trophoblast cells, where it seems to exert a protective role in sustaining immune tolerance between the fetus and the mother during pregnancy (8, 12, 13). Soluble forms of HLA-G (sHLA-G) may exert a regulative and protective role in both normal conditions (14) and disease. sHLA-G was found able to trigger apoptosis of cytotoxic CD8 cells (15, 16) and to shape T-cell phenotype toward regulatory phenotypes (17–19). Promising studies suggested sHLA-G as tolerogenic in MS pathogenesis: higher levels of sHLA-G in the cerebrospinal fluid (CSF) of patients with MS were positively correlated with less inflammation and no disease activity at MRI detection (7, 20, 21), whereas lower serum sHLA-G levels were found in patients with MS having clinically active disease (22).

The human leukocyte antigens G (HLA-G) gene is located on chromosome 6 in the major histocompatibility complex (MHC) locus (23). The expression of HLA-G protein is inherently affected by the genetic polymorphism characterizing the gene locus. The main polymorphisms that regulate the HLA-G production are: (i) a deletion/insertion of 14 base pairs (14 bp) and (ii) a single-nucleotide polymorphism (SNP) where a cytosine substitutes guanine (C>G) at the position +3142 bp in the untranslated region at the 3' of the gene (3' UTR) (23, 24), as demonstrated by Cree and co-authors (24). These polymorphisms impact on mRNA stability *in vivo*: it was demonstrated that the genotypes 14 bp insertion/insertion (ins/ins) and +3142 G/G determine a low production of HLA-G compared to 14 bp insertion/deletion (ins/del) or to the genotypes deletion/deletion (del/del) and +3142 C/G or C/C (20, 25–27). Of note, these functional polymorphisms are associated with MS susceptibility in the Tunisian population (8). Furthermore, the presence of the 14 bpI affects mRNA stability and protein production (27) and is associated with pregnancy pathologies and autoimmune diseases (28, 29). On the other hand, the +3142 G allele binding to 3 microRNAs (miRNAs) miR-148a, miR-148b, and miR-15 is predicted to be more stable than binding to the +3142 C allele, resulting in lower protein production (25). We have previously demonstrated that the highest and the lowest plasma sHLA-G values were identified in patients with MS having +3142 C/C and 14 bp D/D and +3142 G/G and 14 bp I/I genotypes, respectively (30). These findings raised the issue of whether sHLA-G may potentiate the immunomodulant action of MS treatments. A recent study reports a higher serum sHLA-G level in patients with MS under interferon-β compared to healthy individuals, although no differences were found between the overall MS cohort and healthy people (31).

Despite these interesting findings, our knowledge about the role of different HLA-G genotypes in MS pathogenesis and in MS treatment outcomes is still very limited. With the aim to shed light on this topic, we investigated sHLA-G and its genotypes in the serum of patients with RRMS before and after 12 and 24 months of treatment with natalizumab (NTZ), a monoclonal antibody that blocks the alpha4-integrin, or very late antigen-4 (VLA-4), expressed on the surface of T lymphocytes, preventing their migration into the CNS. NTZ is an effective second-line immunomodulant treatment for RRMS, usually applied when first-line treatments fail. NTZ is known to impact immune cell populations, especially leading to a reversible increase of peripheral cell counts due to its mechanism of action (32, 33). With this study, we showed that patients with RRMS are distinguishable in different subgroups based on their serum sHLA-G concentration. Furthermore, when under NTZ, most high and medium sHLA-G producers were free from MRI activity after 24 months of treatment.

## Materials and Methods

### Patients Enrollment: Characteristics and Inclusion Criteria

A total of 27 patients were enrolled at the Department of Neurology II at Careggi University Hospital, Florence, Italy. Patients were diagnosed with RRMS according to McDonald criteria (34) and shared the following characteristics: age between 18 and 60 years; Expanded Disability Status Scale (EDSS) score between 0 and 5.5. Inclusion and exclusion criteria are described in detail in SURPASS study (ClinicalTrials.gov Identifier: NCT01058005). Patient characteristics are reported in Table 1. Patients with RRMS included in the study showed highly active disease before NTZ treatment, e.g., failed the first-line treatments or presented rapidly evolving MS with 2 or more relapses in 1 year and 1 or more Gd+ lesions with a significant increment of T2 lesions. At the baseline sample, patients were not receiving any disease-modifying treatments (DMTs).

**Table 1:** Patients' characteristics.

No. of patients	27
F:M ratio	20:7
Age (years) (median, range)	36 (20–52)
Disease duration (months) (average, range)	87.53 (2–188)
EDSS at baseline (T0) (median, range)	2 (1–4)
EDSS at T12 of NTZ (median, range)	1.5 (0–6)
EDSS at T24 of NTZ (median, range)	1.8 (1–6)
ARR^A^ last 2 years	2 (0–4)
ARR last year median (range)	1 (0–4)
**Treatments pre-NTZ**	
IFN^B^ (n. of administration) (average, range)	55.75 (5–158)
Cop^C^ (n. of administration) (average, range)	39.67 (9–94)
AZA^D^ (n. of administration) (average, range)	55.22 (9–94)

A *ARR, analyzed relapse rate;*

B *IFN, interferon-β 1a;*

C *Cop, Copaxone;*

D *AZA, Azathioprine. In this study, before NTZ 23 patients were treated with IFN, 5 with Cop, 13 with AZA and 4 did not receive any treatment*.

### Study Approval and Patient Consents

The study was performed according to the Declaration of Helsinki. Written informed consent was signed by all patients involved in the study, and approved by the Local Ethical Committee (#CEAVC12745).

### Patients' Clinical Follow-Up

During NTZ, patients with RRMS underwent clinical follow-up every 6 months and at least yearly brain MRI, according to clinical practice. All patients with RRMS were evaluated for the presence of anti-JCV and anti-NTZ antibodies in the serum. Clinical MRI and laboratory data were retrospectively collected; occurrence of relapses and MRI activity (new T2 lesions and/or Gd+ lesions) were recorded. Disability was assessed by EDSS score. Additional clinical evaluations were performed upon patients' request in the event of new neurological symptoms or any other neurological issues.

### Peripheral Blood and Serum Collection

For each patient, the whole peripheral blood (PB) was collected in heparin-containing tubes. Peripheral blood mononuclear cells (PBMCs) were collected by density gradient centrifugation by Pancoll (density: 1.077 g/ml, PAN-Biotech) at 1,500 rpm, RT, for 30 min within 2 h from PB collection. To collect serum, 10 ml of blood were drawn in serum-separated tubes (SSTs) according to the same time schedule as performed for PB. Serum was then separated from blood by centrifugation at 3,000 rpm, RT, for 10 min, then aliquoted and stored in −80°C freezer until used.

### Patients Immunophenotype

Immunophenotype of patients with RRMS during NTZ was analyzed as routine from fresh whole blood upon erythrocyte lysis (BD Lysis buffer, BD Bioscences) by labeling cells with the following panel of fluorescent monoclonal antibodies: anti-CD3 FITC (clone: clone SK7), anti-CD16 PE clone B73.1 and anti-CD56 PE (clone NCAM16.2), anti-CD45 PerCP-Cy5.5 (clone 2D1), anti-CD4 PE-Cy7 (clone: SK3), anti-CD19 APC (clone: SJ25C1), and anti-CD8 APC-Cy7 (clone: SK1). Data acquisition was performed using a 3-laser 8-color flow cytometer (FACSCanto II, BD Biosciences); data were analyzed using FACSDiva software version 8.0.1. (BD Biosciences).

### Serum Soluble HLA-G Analysis

The concentration of sHLA-G in serum samples was measured in triplicate by ELISA as previously described (30, 35, 36) by using the capture monoclonal antibody (MoAb) MEM-59 (Exbio, Praha, Czech Republic), which recognizes the β2-microglobulin-associated form of HLA-G. The intra- and inter-assay coefficient of variation (CV) was 1.4 and 4%, respectively. The sensitivity limit was 1.0 pg/ml.

### HLA-G Polymorphism Typing

Genomic DNA (gDNA) was isolated from PB by the Nucleon Bacc 3 kit (Amersham Pharmacia Biotech, Buckinghamshire, UK) according to the manufacturer's protocol. The HLA-G polymorphism 14 bp ins/del was genotyped by PCR as previously described (30). Briefly, 100 ng of gDNA were amplified in a 25 μl reaction together with 10 pmol of each primer (GE14H LAG, RHG4). The HLA-G polymorphism +3142 C>G was genotyped by the 7300 Real-Time PCR System (Applied Biosystems) using a forward primer 3142 for (50-CCTTTAATTAACCCAT-CAATCTCTCTTG-30), a reverse primer 3142 rev (50-TGTCTCCGTCTCTGTCTCAAATTT-3), and 2 probes for the identification of the 3142 C (0-VIC-TAAGTTATAGCTCAGTGGAC-30; 3142CFVIC) or the 3142G (50-FAM-TAAGTTA-TAGCTCAGTGCAC-30; 3142GFAM) allele, respectively.

### Cytokines and Chemokines Measurement

Serum from each patient was analyzed by Bioplex device (Biorad) using Milliplex assay (Merck Millipore) following the manufacturer's protocol for the determination of the following 17 cytokines and chemokines: IL1α, IL1β, IL2, IL4, IL6, IL8, IL10, IL12p40, IL12p70, IL17, IL23, IFNγ, TNFα, GM-CSF, CXCL10, CXCL13, and MMP9. Cytokines and chemokines concentrations were reported in pg/ml. The sensitivity limit was 1.0 pg/ml.

### Statistics

Statistical analyses were performed by IBM SPSS statistic 20 (IBM Corp) and by GraphPad Prism v.6 (GraphPad Software, Inc.). One-way ANOVA followed by *post-hoc* Tukey's test was used to compare patient subgroups (high, medium, and low sHLA-G producers) for peripheral absolute cell counts, serum cytokines level, HLA-G genotype distribution, and sHLA-G level over 24 months of NTZ treatment. Non-parametric Kaplan–Meier estimator or chi-square test were used to compare patient subgroups for MRI disease activity by evaluating the cumulative proportion of survival percentage. Pearson correlation coefficient (*r*) was calculated to evaluate the correlation between variables. Statistical significance was considered when *p*-value < 0.05.

## Results

### Absolute Peripheral Cell Counts of RRMS Cohort Increase, Along With Serum Level of IL2 and TNFα, Over Natalizumab Treatment

**Figure 1:**
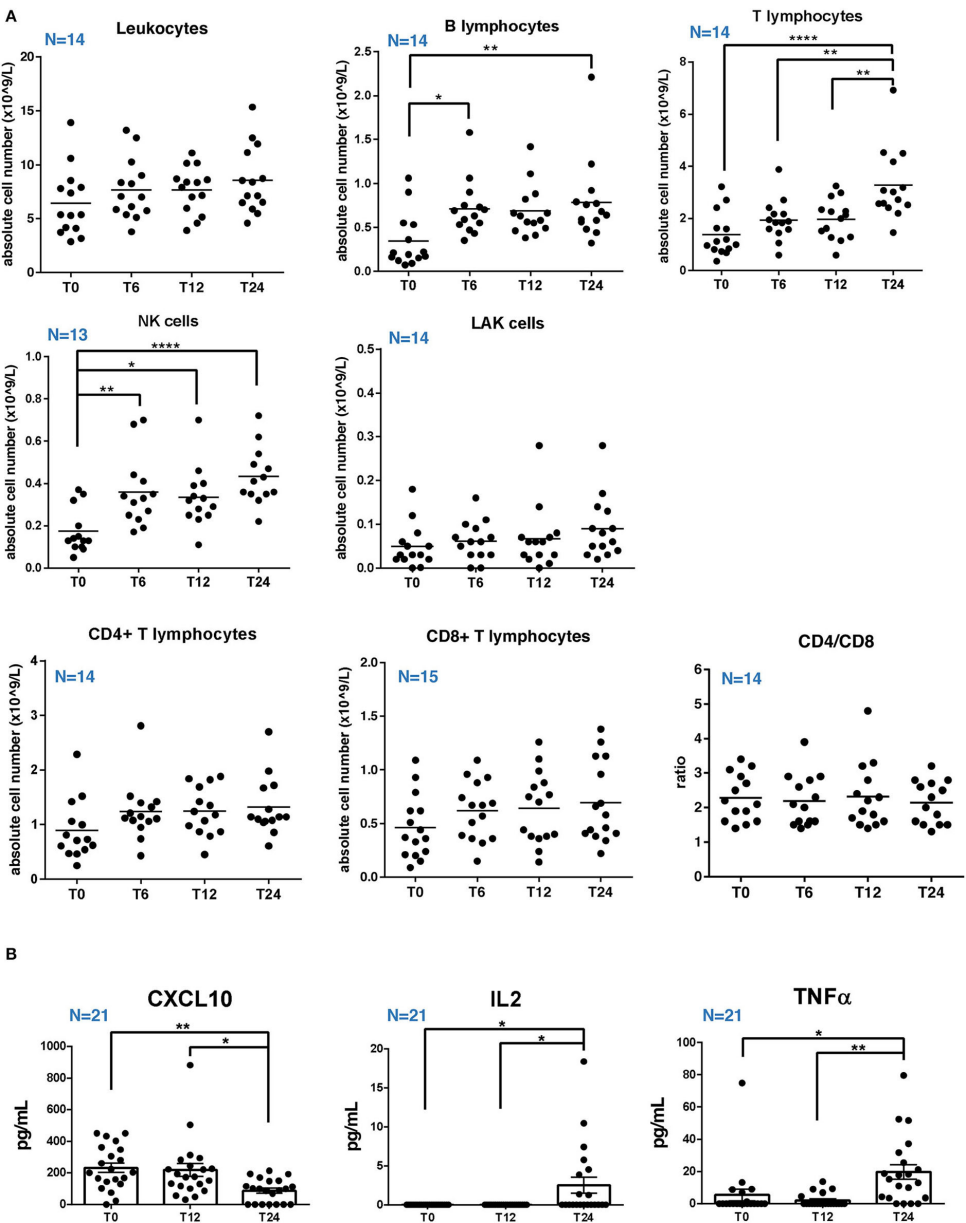
Absolute cell counts of circulating lymphocytes and serum cytokines level in RRMS patients under natalizumab. **(A)** Percentage and absolute cell count of total T, B, natural killer (NK), lymphokine-activated killer (LAK) cells, and the ratio between CD4 and CD8 T cells (CD4/CD8) at baseline (T0) and after 6, 12, or 24 (T6, T12, and T24) months of natalizumab (NTZ) treatment. Each dot represents a patient. Patients' number is shown in blue for each graph. Mean ± SEM is reported. Statistical significance was determined by one-way ANOVA followed by *post-hoc* Tukey test (*p < 0.05; **p < 0.01; and ****p < 0.0001). **(B)** Concentration (pg/ml) of CXCL10, TNFα, and IL2 in serum of patients with RRMS at T0 or at T12 and T24 of NTZ treatment. Each dot represents a patient. Patients' number is shown in blue for each graph. Mean ± SEM is reported. Statistical significance was determined by one-way ANOVA followed by *post-hoc* Tukey test (*p < 0.05; **p < 0.01).

Immunophenotypes were analyzed by flow cytometry on fresh blood samples of patients. Phenotypic analyses were performed before starting the treatment (T0) and at 6, 12, and 24 months of NTZ treatment (T6, T12, and T24) and included the evaluation of the percentage and absolute cell count of total T, B, natural killer (NK), lymphokine-activated killer (LAK) cells, and CD4/CD8 ratio. Flow cytometry gating strategy and analysis of cell subsets are reported in Supplementary Figure 1. Accordingly to the mechanism of action of NTZ, which blocks immune cells in the periphery, we found an increase in absolute cell counts over time (Figure 1A). Such increase is significant in B lymphocytes at T6 (*p*-value = 0.04) and T24 (*p*-value = 0.01) compared to T0; in T lymphocytes at T24 compared to T0 (*p*-value < 0.0001), T6 (*p*-value = 0.003), and T12 (*p*-value = 0.004); and in NK cells at T6 (*p*-value = 0.007), T12 and T24 (*p*-value < 0.0001) compared to T0. The increase of total leukocytes and of CD4 and CD8 absolute counts over time was not statistically significant. CD4/CD8 ratio was not altered. On serum of patients with RRMS, we determined the level of 17 cytokines and chemokines at T0, T12, and T24 of NTZ, to evaluate fluctuations in the peripheral inflammatory profile of patients over treatment. In our analysis, IL1α, IL1β, IL2, IL17, IFNγ, IL10, and GM-CSF were below the detection limit (1.0 pg/ml) at all time points, therefore excluded from the analysis. CXCL10, TNFα, and IL2 showed significant variation over time: specifically, we found a significant decrease of CXCL10 at T24 compared to T0 (*p*-value = 0.004) and T12 (*p*-value = 0.01); a significant increase of TNFα at T24 compared to T0 (*p* value = 0.01) and T12 (*p*-value = 0.001) and a significantly higher level of IL2 at T24 compared to T0 and T12 (*p*-value = 0.01) (Figure 1B). To sum up, T, B, and NK cells significantly augmented over 24 months of NTZ treatment, along with serum levels of TNFα and IL2.

### RRMS Patients Under Natalizumab Are Distinguishable Into High, Medium, and Low sHLA-G Producers

We evaluated the levels of sHLA-G in serum samples of 27 patients with RRMS at baseline (T0) and at T12 and T24 of treatment with NTZ. Based on sHLA-G concentration (ng/ml), we considered patients as low producers with a serum sHLA-G level below 10 ng/ml at T0 and as high producers with a serum sHLA-G level up to 20 ng/ml at T0. With an sHLA-G level between 10 and 20 ng/ml, patients were classified as medium producers. Therefore, the 27 patients with RRMS were subdivided into 6 high sHLA-G producers, 7 medium producers, and 14 low producers.

**Figure 2:**
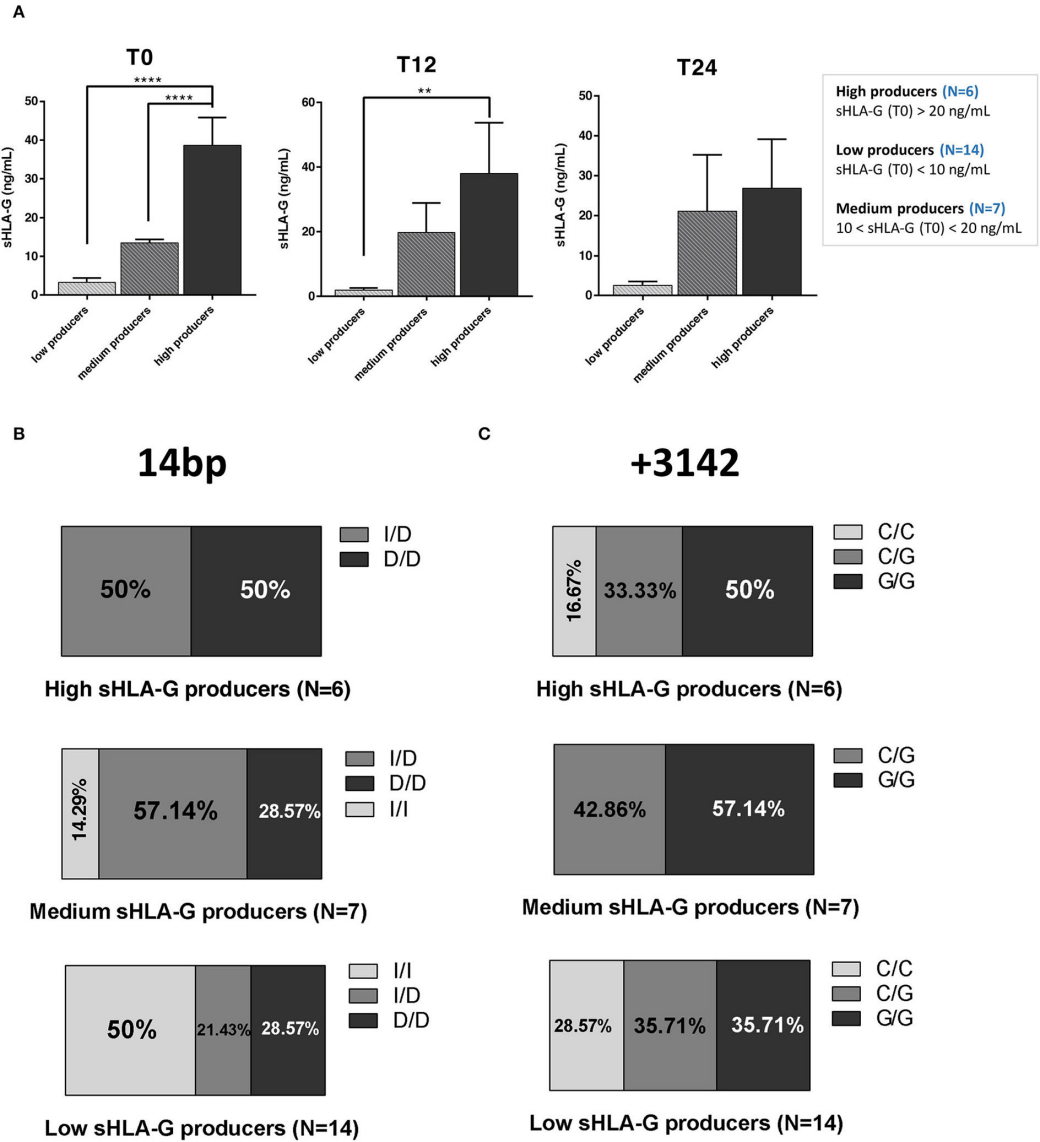
RRMS patients under natalizumab are divided into high, medium, and low sHLA-G producers, who are characterized by different 14 bp and +3142 genotypes distribution. **(A)** sHLA-G production (ng/ml) in low, medium, and high producers at baseline (T0; left graph), at T12 (middle graph), and at T24 (right graph) of NTZ treatment. A total of 27 RRMS were divided into 3 groups (14 low producers, 7 medium producers, and 6 high producers) based on serum sHLA-G concentration, as reported in the legend. Mean ± SEM is reported (one-way ANOVA followed by *post-hoc* Tukey's test; **p < 0.01; ****p < 0.0001). **(B)** Percentage of 3 different genotypes of the 14 bp polymorphism of the HLA-G gene in high, medium, and low sHLA-G producers within the RRMS cohort: insertion/insertion (I/I), insertion/deletion (I/D), and deletion/deletion (D/D). **(C)** Percentage of 3 different genotypes of the +3142 C>G polymorphism (C/C, C/G, and G/G) of the HLA-G gene in high, medium, and low sHLA-G producers within the RRMS cohort. Chi-square test was used.

We found that the serum sHLA-G level is significantly (*p*-value < 0.0001) higher in high producers compared to low and medium producers at baseline (T0) (Figure 2A). At T12, sHLA-G is significantly (*p*-value = 0.006) increased in high producers compared to low producers. No significant differences were detected between groups at T24, and across timepoints within each group (high producers: mean ± SEM at T0 = 38.65 ± 7.24 ng/ml; mean ± SEM at T12 = 38.06 ± 15.68 ng/ml; mean ± SEM at T24 = 26.86 ± 12.25 ng/ml; medium producers: mean ± SEM at T0 = 13.49 ± 0.88 ng/ml; mean ± SEM at T12 = 19.74 ± 9.21 ng/ml; mean ± SEM at T24 = 21.08 ± 14.23 ng/ml; low producers: mean ± SEM at T0 = 3.36 ± 1.07 ng/ml; mean ± SEM at T12 = 1.86 ± 0.79 ng/ml; and mean ± SEM at T24 = 2.61 ± 0.91 ng/ml).

### 14 bp and +3142 Genotypes Are Differentially Distributed Among High, Low, and Medium sHLA-G Producers

We next genotyped patients with RRMS and divided them according to the results. The group including the 14 bp HLA-G genotype was subdivided based on the polymorphism into ins/ins (I/I), ins/del (I/D), and del/del (D/D) subgroups. The group including the +3142 genotype was subdivided based on the SNP C>G in C/C, C/G, and G/G subgroups.

Globally, we did not find any significant difference in polymorphism distribution among high, medium, and low sHLA-G producers. High producers (*N* = 6) are equally divided between patients carrying the 14 bp I/D and D/D polymorphism. Medium producers (*N* = 7) are mainly represented by the 57.14% of patients carrying the 14 bp I/D genotype, followed by the 28.57% with the D/D genotype and the remaining 14.29% with the I/I one. On the other hand, the 50% of low producers (*N* = 14) were characterized by the 14 bp I/I genotype, whereas the 21.43% and the 28.57% with the 14 bp I/D and D/D genotypes, respectively (Figure 2B). Concerning +3142 genotypes (Figure 2C), half of the high producers carry the G/G genotype (50%), whereas the other half is divided between C/G (33.33%) and C/C (16.67%) genotypes. Medium producers were typed as C/G (42.86%) or G/G (57.14%); none of them was typed as C/C. Finally, low producers are equally distributed between C/G and G/G genotypes (35.71%), with 28.57% carrying the C/C genotype.

In summary, genotype polymorphism distribution does not significantly differ among sHLA-G producers; of note, 14 bp I/I genotype is not present among high producers, and +3142 C/C among medium producers.

### Serum sHLA-G Variation in RRMS Patients Over 24 Months of Natalizumab

To investigate a possible correlation between treatment/genotype and sHLA-G serum level, we evaluated the sHLA-G production during NTZ treatment and the associated genotype distribution.

**Figure 3:**
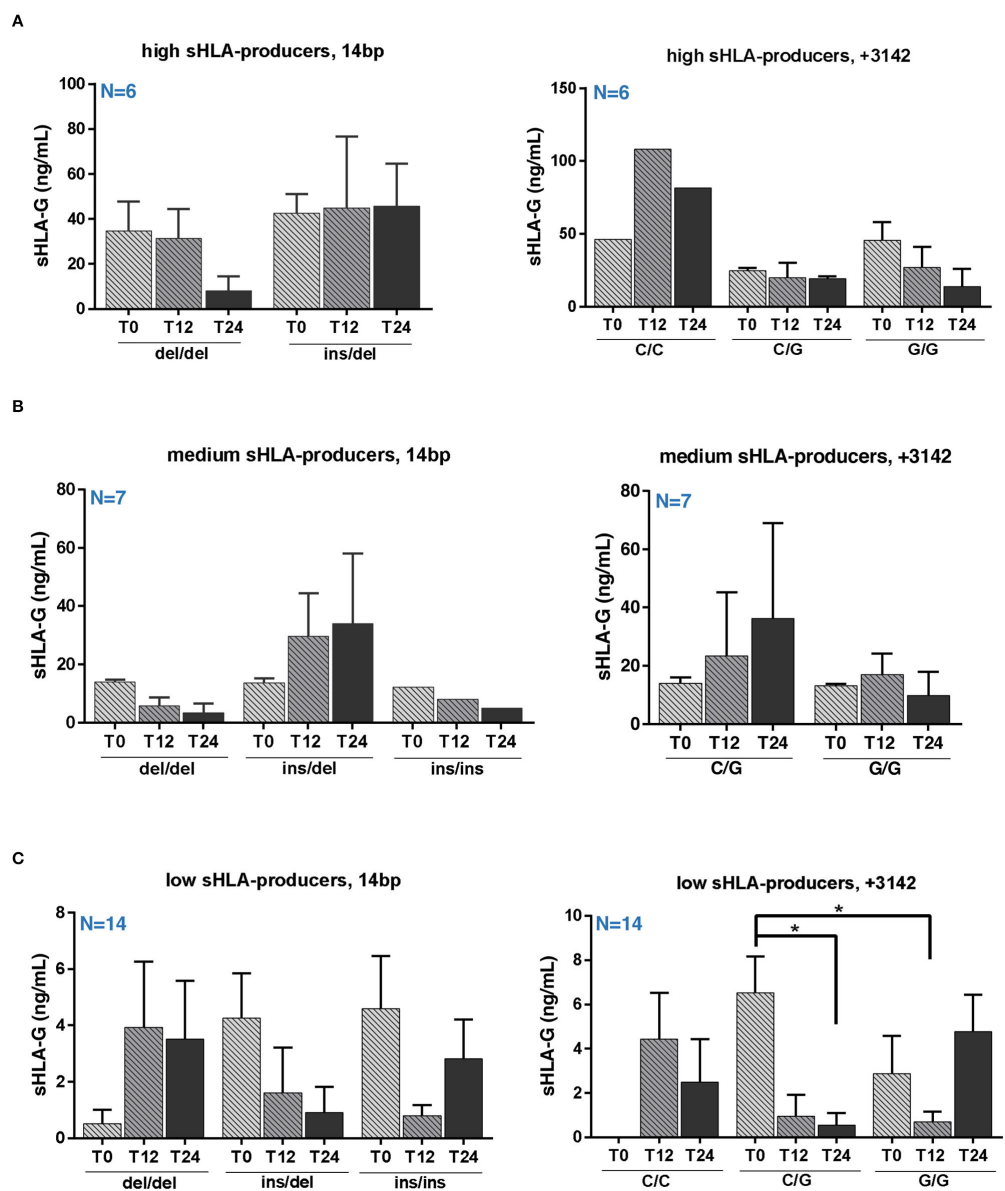
Serum sHLA-G variation in RRMS patients during natalizumab is partially genotype-dependent. Serum sHLA-G production (ng/ml) in RRMS patients at T0, T12, and T24 of NTZ treatment divided patients into high **(A)**, medium **(B)**, and low **(C)** sHLA-G producers. Each group of patients is furtherly distinguished based on their HLA-G genotype (14 bp, left graphs; +3142 bp, right graphs) and polymorphisms. Patients' number is shown in blue for each graph. Mean ± SEM is reported; only the mean is shown for groups including a single observation. Statistical significance was determined by one-way ANOVA followed by *post-hoc* Tukey test (*p < 0.05).

We did not find any significant variation in sHLA-G production over NTZ treatment among high and medium producers based on 14 bp (Figures 3A,B, left panels) or +3142 (Figures 3A,B, right panels) polymorphisms. Of note, sHLA-G production is quite stable among +14 bp I/D high producers over 24 months of treatment (mean ± SEM at T0 = 42.65 ± 8.46 ng/ml; mean ± SEM at T12 = 44.80 ± 31.82 ng/ml; and mean ± SEM at T24 = 45.64 ± 18.87 ng/ml) (Figure 3A, left graph). On the other hand, sHLA-G production is globally variable among medium producers (Figure 3B). Concerning low producers, we observed a significant (*p* = 0.032) decrease of sHLA-G at T24 compared to T0 in patients carrying the +3142 C/G genotype and a significant (*p* = 0.038) decrease at T12 of +3142 G/G patients compared to C/G at T0 (Figure 3C). Of note, we did not find any positive correlation, calculated as Pearson coefficient, between sHLA-G levels and peripheral absolute cell counts of patients or with respect to serum cytokines (data not shown).

### The Majority of High and Medium Producers Are Free From MRI Activity After 24 Months of Treatment

**Figure 4:**
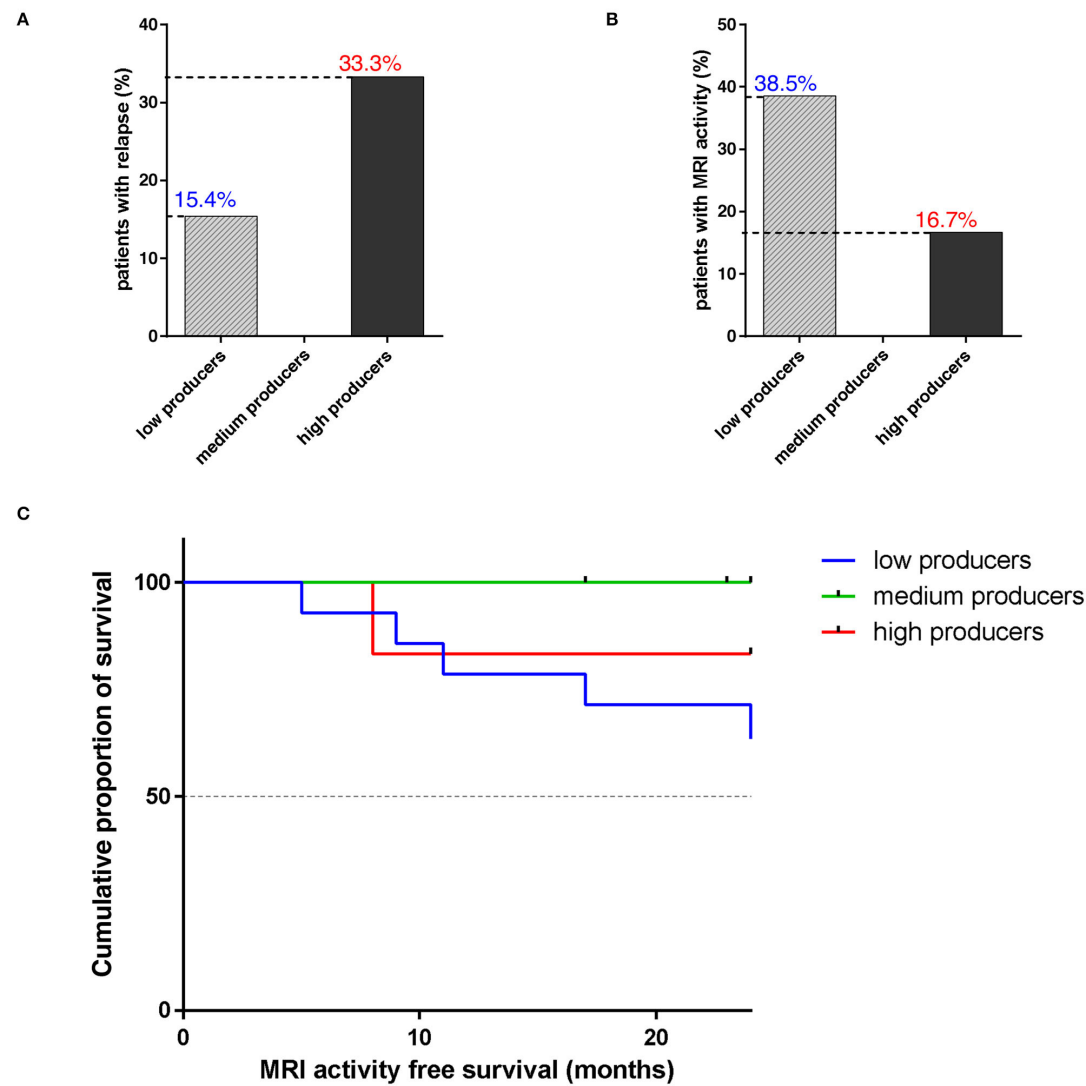
The majority of high and medium sHLA-G producers are free from disease relapse and MRI activity after 24 months of natalizumab. **(A)** Percentage of patients with RRMS, divided into low (*N* = 13; 1/14 was lost from follow-up at 17 months), medium (*N* = 6; 1/7 was lost from follow-up at 17 months), and high (*N* = 6) sHLA-G producers, who experienced a disease relapse during NTZ treatment. Chi-square test was used. **(B)** Percentage of patients with RRMS, divided into low, medium, and high sHLA-G producers, who showed MRI disease activity during treatment. **(C)** Cumulative proportion of survival (percentage) from MRI activity of patients with RRMS over NTZ treatment (time from 0 to 24 months is reported on x-axis) for low (blue line), medium (green line), and high (red line) sHLA-G producers. Chi-square test was used.

The occurrence of relapses is rare during NTZ treatment (37). In our sample, among high producers, 2/6 (33.3%) experienced a relapse; among medium producers, 1/7 was lost from follow-up at 17 months and no one of the other 6 patients experienced a relapse. On the other hand, 2/13 (1/14 was lost from follow-up at 17 months) low producers relapsed (15.4%) (Figure 4A). Patient MRI shows that 5/13 low producers had new T2 lesions and, among them, 2/5 had Gd-enhancing lesions at follow-up scan. Among these, 2 had a relapse. Concerning other groups, only 1 high producer showed Gd-enhancing lesions or T2 new lesions (Figure 4B). Differences among producers were not statistically significant.

Magnetic resonance imaging (MRI) activity-free survival was 83.3% in high sHLA-G producers, 63.5% in low producers, and 100% in medium producers; such difference was not statistically significant (*p*-value = 0.211) (Figure 4C).

## Discussion

In this work, we evaluated the sHLA-G production and genotype in patients with RRMS during NTZ treatment. We measured serum sHLA-G level and correlated this feature with treatment outcome in terms of disease relapses and MRI activity. Our data suggest that low sHLA-G producers are more at risk of showing disease activity during treatment compared to high and medium producers.

Soluble forms of HLA-G (sHLA-G) molecule is known for contributing to maintaining the immune tolerance in both health and disease (15) and has been correlated to a better disease outcome when high in CSF of patients with MS (6). Here, we were able to divide our RRMS cohort into 3 groups, low, medium, and high producers, based on their serum sHLA-G level. Within each group, we did not observe any significant variation in sHLA-G level; instead, we found that sHLA-G concentration is significantly higher in high producers compared to low and medium producers at baseline and with respect to low producers at T12 (Figure 2A).

The characterization of patients by sHLA-G genotype revealed that I/D and D/D genotypes are mostly represented in high and medium producers, while I/I genotype is not present among high producers (Figure 2B), in accordance with the role of this genotype in controlling HLA-G production (30, 31). In fact, the 14 bp I allele affects mRNA stability and protein production, with the consequent lower secretion of sHLA-G.

When investigating sHLA-G concentration across patients based on their genotypes, we did not observe any significant variation among high and medium producers (Figures 3A,B), whereas +3142 C/G low producers reported a significant decrease of sHLA-G after 24 months of NTZ compared to baseline (Figure 3C).

The analysis of serum cytokines and chemokines in the RRMS cohort showed a significant decrease in CXCL10 at T24 and a significant increase in TNFα and IL2 at T24 (Figure 1B). The increase in cytokines level is expected during NTZ, since the treatment leads to a peripheral enrichment of T cells, including potential pathogenic clones, which is also in agreement with the immunophenotype of patients showing a significant increase in the absolute cell counts over treatment (Figure 1A). Comparing these results with sHLA-G variation over time, we did not observe any association between sHLA-G changes and either cytokines or absolute cell counts variations; therefore, we suggest that sHLA-G production does not depend on the numerousness or function of T cells in peripheral blood.

We then evaluated the association between serum sHLA-G production and patients' outcomes to NTZ treatment in terms of relapse rate and MRI disease activity over 24 months (Figure 4). Results showed that 33.3% of high, 15.4% of low, and none among medium producers experienced a relapse (Figure 4A). On the other hand, 38.5% of low sHLA-G producers, compared to medium (0%) and high (16.7%) showed MRI disease activity (Figure 4B). Finally, 83.3% of high and 100% of medium producers were MRI-activity free at T24, with respect to 63.5% of low producers (Figure 4C).

### Study Limitations and Conclusions

To sum up, we found that sHLA-G significantly decreases over NTZ treatment in low sHLA-G producers carrying the +3142 C/G genotype (Figure 3C) and that serum sHLA-G concentration does not correlate with peripheral cell counts or peripheral inflammatory profile. Our findings also showed a variable distribution of HLA-G polymorphisms among producers (Figures 2B,C) and, finally, 83.3% of high and 100% of medium producers are free from MRI activity over 24 months of treatment, with respect to 63.5% of low producers (Figure 4C). Although interesting, these differences between groups are not statistically significant: the narrowness of our 27-RRMS patient cohort is in fact the main limitation of our study and may have impacted on significance. However, in light of numerous previous findings pointing out sHLA-G's role in modulating immunotolerance in MS (6, 21, 22, 24, 30, 35, 36), we suggest that sHLA-G contributes to NTZ treatment outcome in patients with RRMS and that such contribution would be particularly noticeable when considering MRI activity; therefore, we stress the need for further confirmation by larger cohort size.

## Data Availability Statement

The raw data supporting the conclusions of this article will be made available by the authors, without undue reservation.

## Ethics Statement

The study was performed in accordance with the Declaration of Helsinki, reviewed and approved by the Local Ethics Committee of the Tuscany region, Centre Area (#CEAVC12745). The patients/participants provided their written informed consent to participate in this study.

## Author Contributions

RA experiments (PBMCs and serum collection, DNA isolation, and cytokines assay), manuscript writing, and data analysis. RR, DB, and VG experiments (patients HLA-G typization and sHLA-G quantification) and data analysis. AM manuscript writing and clinical data analysis. EB and AA PBMCs and serum collection, cytokines assay, and data analysis. AC data analysis. BP flow cytometry analysis of patients' immunophenotype. AR clinical data collection. LM critical discussion on clinical data. EF MRI data collection. CB study conceptualization and manuscript writing revision. All authors contributed to the article and approved the submitted version.

## Funding

This work was supported by FISM 2019 grant code 2019/R-Single/004.

## Conflict of Interest

The authors declare that the research was conducted in the absence of any commercial or financial relationships that could be construed as a potential conflict of interest.

## Publisher's Note

All claims expressed in this article are solely those of the authors and do not necessarily represent those of their affiliated organizations, or those of the publisher, the editors and the reviewers. Any product that may be evaluated in this article, or claim that may be made by its manufacturer, is not guaranteed or endorsed by the publisher.
