# Supplementary material for: Investigating Serum sHLA-G Cooperation With MRI Activity and Disease-Modifying Treatment Outcome in Relapsing-Remitting Multiple Sclerosis
Source: Front Neurol. 2022 May 25;13:872396. doi: 10.3389/fneur.2022.872396 (PMC9174986; doi:10.3389/fneur.2022.872396)
Supplement: Supplementary file 1 [file Image_1.pdf]

Supplementary Figure 1

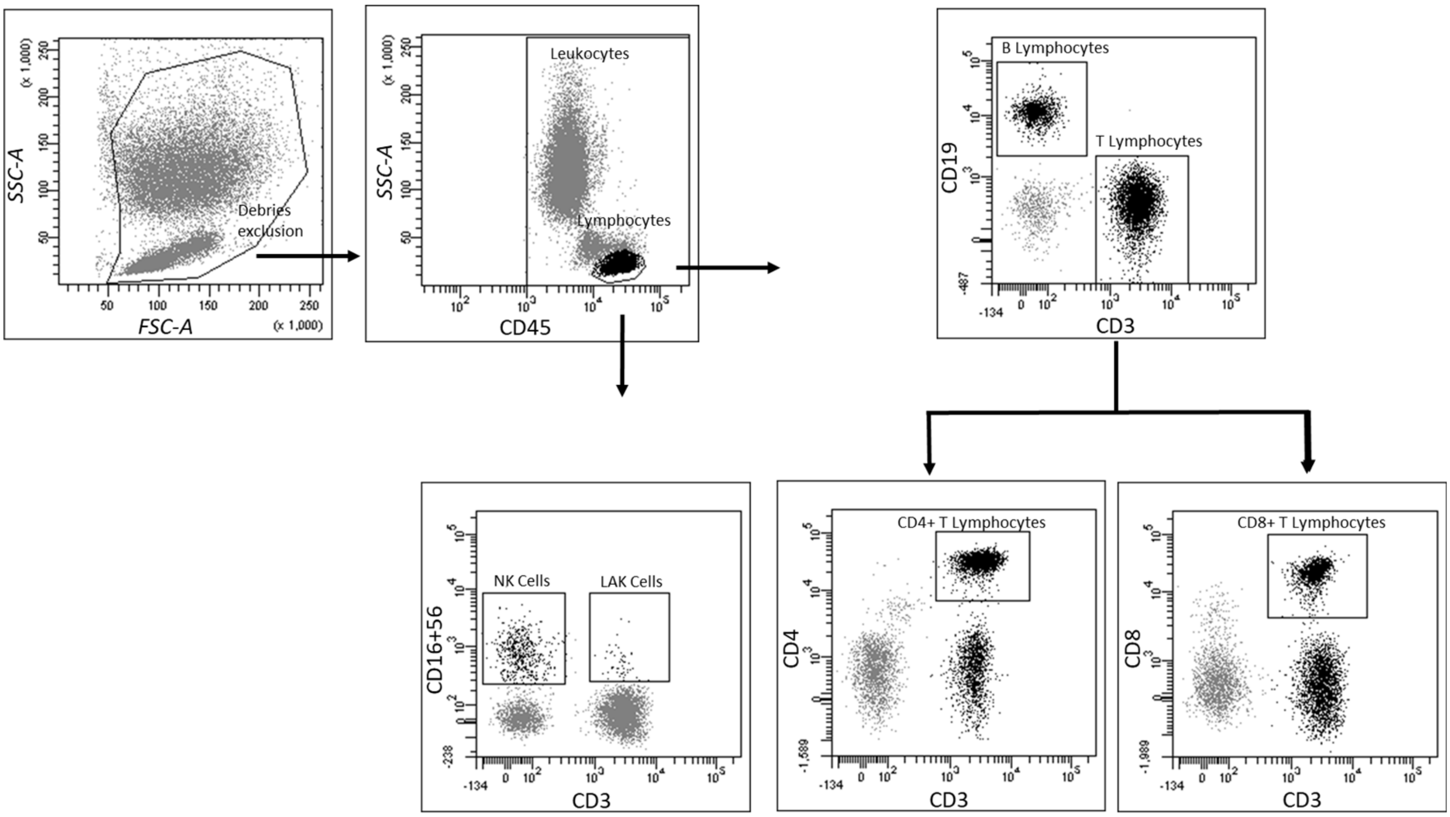

Flow cytometry gating strategy for lymphoid cell subsets immunophenotyping of RRMS patients.
Representative dot plots of the flow cytometry gating strategy for lymphoid cell subsets identification from fresh whole blood of RRMS patients. Debris were removed in a FSC-A *vs* SSC-A parameters and total lymphocytes were identified in a CD45 *vs SSC* scatter. B cells were identified as CD19+ and T cells as CD3+ in a CD19 vs CD3 scatter plot. We then identified CD4+ and CD8+ T cells selected in CD4 *vs* CD3 and CD8 *vs* CD3 plots, respectively. Natural Killer (NK) cells (CD3-/CD16+CD56+) were also identified in a CD3 vs CD16+CD56+ scatter plot as well as Lymphokine-activated killer (LAK) cells (CD3+/CD16+CD56+). All the analyses were performed using a FACSCanto II flow cytometer (BD Biosciences, San Jose, CA); data were analyzed using FACSDiva software version 8.0.1.
